# Pre-treatment T cell features and immune-milieu characteristics shape treatment-induced exhaustion and resistance to Blinatumomab in B-cell acute lymphoblastic leukemia

**DOI:** 10.3389/fimmu.2026.1895742

**Published:** 2026-08-20

**Authors:** Francesco Corrado, Viktoria Blumenberg, Maryam Kazerani, Jan Wulf, Tobias Straub, Nora Philipp, Daniel Nixdorf, Amelie Muth, Giulia Rappa, Agnese Petrera, Michaela Scheurer, Chang-Feng Chu, Giulia Magno, Christina E. Zielinski, Michael von Bergwelt-Baildon, Marion Subklewe, Veit Bücklein

**Affiliations:** 1 Laboratory for Translational Cancer Immunology, LMU Gene Center, Munich, Germany; 2 Department of Medicine III, LMU University Hospital, Ludwig Maximilian University (LMU) Munich, Munich, Germany; 3 Hematology and Cellular Therapy, Department of Medicine, Cedars-Sinai Medical Center, Los Angeles, CA, United States; 4 Metabolomics and Proteomics Core Facility, Helmholtz Center Munich - German Research Center for Environmental Health (GmbH), Munich, Germany; 5 TranslaTUM at Technical University of Munich, Munich, Germany; 6 Department of Infection Immunology, Leibniz-Institute for Natural Product Research and Infection Biology, Jena, Germany; 7 Friedrich Schiller University of Jena, Germany & Department of Pathology, University of Cambridge, Jena, United Kingdom

**Keywords:** acute B cell lymphoblastic leukemia, multiomic analyses, proteomic analysis, single cell CITE sequencing, T cell redirecting bispecific antibodies (bsAbs)

## Abstract

**Background:**

Blinatumomab (Blina), a CD19×CD3 bispecific T cell engager, is approved for the treatment of B-cell precursor acute lymphoblastic leukemia (BCP-ALL), yet resistance remains a major challenge and the mechanisms driving treatment failure remain poorly understood.

**Methods:**

To define the immunological determinants of resistance, we performed longitudinal profiling of peripheral blood T cells and the immune milieu of 34 patients receiving Blina using flow cytometry (n=19), single-cell CITE-seq (n=13), *ex vivo* Blina-induced cytotoxicity (n=26) and serum proteomics (n=17).

**Results:**

At baseline, Responders (R) were enriched for CD8_+_ effector memory T cells (T_EM_) expressing higher levels of cytotoxic genes and their transcriptional regulator *ZNF683*. Conversely, CD8^+^ T_EM_ from Non-Responders (NR) displayed transcriptional features of activation without proportionate cytotoxic commitment. Over the course of the first treatment cycle, NR exhibited a progressive expansion of TIM3_+_CD8_+_ T cells that correlated with a rapid loss of *ex vivo* cytotoxic function. Linking baseline state to post-treatment T-cell exhaustion, the magnitude of TIM3_+_CD8_+_ expansion correlated inversely with baseline *ZNF683* expression in CD8_+_T_EM_. Beyond T-cell-intrinsic features, NR harbored an immunosuppressive milieu characterized by higher circulating levels of M2-polarizing factors (CSF-1, HGF) and the TIM-3 ligand Galectin-9, which correlated positively with the magnitude of TIM3_+_CD8_+_ T-cell expansion.

**Conclusions:**

These findings indicate that post-Blina CD8^+^ T-cell exhaustion is associated with resistance and it is shaped by both reduced *ZNF683*-dependent cytotoxic programming in CD8_+_ T_EM_ and an immunosuppressive milieu. This provides a rationale for risk stratification based on baseline transcriptional profiling of CD8_+_ T_EM_ and for combinatorial strategies targeting the suppressive microenvironment.

## Introduction

Blinatumomab (Blina) is a CD19xCD3 bispecific T cell engager that redirects CD3^+^ T cells towards CD19^+^ target cells. It has been approved for the treatment of patients with Relapsed/Refractory (R/R) or Measurable Residual Disease (MRD) positive B-cell Precursor Acute Lymphoblastic Leukemia (BCP-ALL), and more recently in consolidation independent of MRD status (1). Compared to standard of care, Blina confers a significant survival benefit in R/R patients (2), and induces high rates of MRD negativity in patients with MRD^+^ BCP-ALL (3). However, primary resistance rates are close to 50% and 20% in the R/R and MRD^+^ settings, respectively, and the majority of responding patients relapse early, particularly in the R/R setting (median duration of remission 7.3 months (2) in R/R disease, and median relapse-free survival 24 months (3) in MRD^+^ patients). Apart from baseline leukemia burden, determinants of treatment failure are poorly understood, hindering the development of response prediction models and limiting therapeutic strategies to counteract treatment resistance.

Mechanisms of resistance to bispecific T cell engaging antibodies can be broadly classified into tumor-intrinsic and -extrinsic factors. Loss of CD19 expression by BCP-ALL blasts, as frequently seen after CD19-CAR T cell therapy for ALL (4, 5), is only observed in a limited fraction of relapsed cases (8-35%) after Blina (6–8). Increased levels of regulatory T cells (9), exhausted CD8^+^ T cells (10), and PD-L1 expression on tumor cells (11, 12) have been associated with inferior outcomes, though conflicting evidence exists, possibly due to heterogeneity in study designs and patient populations. We previously described that the continuous administration of Blina in patients with BCP-ALL drives T-cell exhaustion and dysfunction (13). However, it remains unclear which patient-specific features drive subsequent T-cell exhaustion following Blina treatment and ultimately influence clinical response. Here, we hypothesized that the pretreatment T cell state and systemic immune milieu may contribute to shape T-cell fates under sustained CD3 stimulation. To explore this, we integrated results from baseline and dynamic T cell profiling (flow cytometry, CITE-seq), functional *ex-vivo* studies, and longitudinal serum proteomics in a cohort of 34 patients. We demonstrate that Blina Non-Responders display a distinct pre-treatment immune profile characterized by lower expression of the transcription factor *ZNF683* in CD8^+^ Effector Memory T cells and higher circulating serum levels of M2-polarizing cytokines and checkpoint ligands (CSF-1, HGF, Galectin-9). By leveraging this multi-omic approach, we provide a rationale for specific risk-stratification biomarkers and novel combinatorial immunotherapies to overcome Blina resistance.

## Methods

### Patient selection and response classification

Patients with a diagnosis of BCP-ALL who received Blina at LMU University Hospital in Munich between January 2010 and July 2023 were selected if peripheral blood mononuclear cells (PBMCs) or serum samples collected during the first cycle of treatment were available (n=34). In addition to demographic characteristics, we collected clinical data on disease stage and treatment response with approval by the local institutional review board (Nos. 216–08 and 19-817). All patients provided written informed consent in accordance with the Declaration of Helsinki. The term “Responders” (R) refers to patients with morphological disease (≥5% blasts in the bone marrow) at treatment start who achieved a complete remission (CR) or CR MRD^+^ patients at treatment start who achieved MRD negativity after the first cycle of treatment. The remaining patients were classified as “Non-Responders” (NR).

### Multiparametric flow cytometry

Multiparametric flow cytometry (MPFC) was performed before treatment start and every 7(+/-3) days during the first treatment cycle. PBMCs from 19 patients were stained with AquaLiveDead, antibodies against T cell (CD45, CD3, CD4, CD8) and memory markers (CCR7, CD45-RA). Anti-PD-1 antibodies were used for all patients, whereas anti-LAG3 and anti-TIM3 were introduced in 2021; their expression was assessed in 12 patients. In each sample, we assessed the proportions of CD4^+^ and CD8^+^ cells with a naïve (CCR7^+^CD45RA^+^), central memory (CCR7^+^CD45RA^-^), effector memory (CCR7^-^CD45RA^-^), and effector memory with CD45RA expression (T_EMRA_, CCR7^-^CD45RA^+^) phenotype. In a subset of 12 patients PBMCs were also stained using phenotypic markers for regulatory T cells (CD25, CD127). All samples were analyzed using Navios (Beckman Coulter, LMU) instruments and FlowJo software (version 10.6.2). Absolute lymphocyte counts (ALC) from routine differential blood counts were used to estimate the number of CD3^+^, CD4^+^, CD8^+^ T cells, memory, and exhausted subtypes. Samples with CD3^+^ T cell counts <30/µL were excluded from downstream analysis.

### Assessment of *ex vivo* T cell function

T cells were negatively isolated from PBMCs of 26 patients using the Pan T Cell Isolation Kit (Miltenyi Biotec, Bergisch Gladbach, Germany) and cocultured with the CD19^+^ ALL cell line REH in culture medium at an effector/target ratio (E:T) of 1:3 and 0.5 ng/mL blinatumomab or control bispecific antibody. After 72 hours, cells were stained with AquaLiveDead and antibodies against CD2, CD4, CD8, and CD19. Blinatumomab-mediated lysis of REH cells was determined using flow cytometry, as previously described (13). Patients with at least one sample available for the analysis at one of the fixed time points (Days 1, 7, 14, 21, 28) were included (n=26, R = 16, NR = 10).

### Single-cell library preparation and data analysis for targeted cellular indexing of transcriptomes and epitopes sequencing

PBMCs from 13 patients were collected on the day of treatment start (days −3 to 0). T cells were enriched via CD3 selection using magnetic-activated cell sorting (MACS). To specifically profile T cells, a custom-conjugated antibody sequencing panel (Ab-seq, n=7, anti-CD8, CD4, CCR7, CD45RA, CD127, CX3CR1, PD1) was employed. A targeted panel, tailored for T cell-related genes (n=477, **Supplementary Table 1**), was utilized to capture the expression profiles of individual T cells. The BD Rhapsody™ Proteomics CITE-Seq platform facilitated simultaneous protein and RNA measurements, enabling comprehensive characterization of the T cell populations at the single-cell level. Libraries were sequenced on NextSeq1000 on P2 flowcells (100 cycles) from Illumina, targeting 8000 reads per cell for transcriptome analysis and 350 reads per Ab-seq antibody per cell for protein expression.

### Clustering and differential gene expression

The raw scRNA-seq data was pre-processed by BD Biosciences using the Rhapsody Analysis pipeline to convert the raw reads into Unique Molecular Identifier (UMI) counts and accurately identify putative cells, as previously described (14, 15). The scRNA-seq and AbSeq counts were loaded and processed with Seurat v. 5.0.0 (16). Low-quality cells were filtered by removing cells expressing less than 25 genes and with less than 50 UMI. These thresholds were empirically applied based on nUMI and nGene distribution at the sample level. Normalization and variance stabilization of scRNA-seq counts were obtained using the Seurat function scTransfom (17). Protein expression data were normalized independently of transcriptomic data using centered log-ratio normalization, where counts were divided by the geometric mean of the corresponding feature across cells, and log-transformed. Detection of highly variable genes showed that all genes for which expression was not null for all cells were considered variable. Dimensionality reduction was performed with principal component analysis (PCA). PC embeddings were corrected for potential batch effects between different sequencing runs using the R package Harmony (18) and the sequencing run identity as the batch covariate. The top 20 harmony components were used to graph neighboring cells using Seurat’s k-nearest neighbors (KNN) function with k=20. Clustering was performed on the neighbor’s graph using Seurat’s shared nearest neighbor (SNN) function at resolution 1.0. Each resulting cluster was then classified based on the expression of canonical genes and surface proteins. The MAST package (19) was employed to identify differentially expressed genes between two groups of cells. Seurat’s AddModuleScore function was used to compute gene signature scores at the single-cell level. Only genes present in the expression matrix were retained for scoring. For sample-level correlation analyses between gene expression and clinical or experimental variables, gene counts were aggregated at the sample level within specific cell type and normalized using DESeq2 (20).

### Multiplexed proteomics assay of serum proteins

Ninety-two human immuno-oncology-related proteins were simultaneously measured using the “Olink immuno-oncology” panel based on Proximity Extension Assay technology (Olink Proteomics AB, Uppsala, Sweden). 17 patients with available serum samples were selected, for 16 of them at least two serum samples were collected at 1 of 5 sequential time points (day 1, 7, 14, 21, 28 of cycle 1). 66 samples were processed across 5 time points, resulting in 6.072 unique observations. Experiments were performed as previously described (21). Assay characteristics and validation data are available from the manufacturer’s webpage (https://olink.com/products-services/target/immune-response-panel/). Data are expressed as Normalized Protein Expression (NPX) units on a log2-scale calculated from normalized Ct values. The Olink^®^ Analyze R-package was used for downstream analysis (22). Samples that did not pass the quality control (n=7) were excluded. Of 66 samples, 59 passed quality control and were included in downstream analysis.

### Statistical analysis

R version 4.3.2 was used for data visualization and statistical analysis. To assess differences in continuous variable distribution between groups, we used the Wilcoxon rank-sum test, and the Benjamini-Hochberg correction was applied to control for false-discovery. Statistical significance was defined as p-value <0.05 with FDR<0.25; all significant p values reported in the manuscript meet these thresholds after adjustment for multiple testing. For our longitudinal monitoring analyses, aimed at uncovering the impact of independent variables on dependent outcomes while accounting for repeated measurements, we employed linear mixed effects models (LMMs). Specifically, separate LMMs were fitted for each T cell subset and each protein using the lme4 package (23), with absolute cell counts and protein NPX values serving as the dependent variables, respectively. In every model, fixed effects for time, response, and their interaction were included, along with a random intercept for patient ID to account for the non-independence of samples from the same patient. The significance of these fixed effects was evaluated via an F-test based on Satterthwaite degrees of freedom and type III sum of squares, as implemented in the lmerTest package (21, 24). Additionally, for the protein analyses, group differences at each time point for proteins passing the FDR threshold were further assessed using a Wilcoxon test.

## Results

### Patient characteristics

A cohort of 34 patients diagnosed with BCP-ALL treated with Blina between 2010 and 2023 were included in the study. Median age was 37 years (range 19-76), 65% (n=22) were males, 14 patients (38%) had overt disease at the time of treatment start (R/R), with a median percentage of bone marrow blasts of 49% (range 6-88%). Nine of these patients (64%) did not achieve complete remission after the first cycle of therapy (NR). The remaining patients received Blina in MRD-positive complete remission (n=20), with five patients (36%; NR) not achieving MRD negativity after the first cycle of Blina (**Table 1**; **Supplementary Figure 1A**, **Supplementary Table 2**).

**Table 1:** Patients’ characteristics.

Variable	Overall	NR	R
n	34	14	20
Age (median [IQR])	36.82 [28.80, 59.02]	37.56 [35.20, 57.83]	34.14 [27.63, 57.95]
Sex = M (%)	23 (67.6)	11 (78.6)	12 (60.0)
Disease Stage = R/R (%)	14 (41.2)	9 (64.3)	5 (25.0)
Previous therapies (%)			
0	2 (5.9)	1 (7.1)	1 (5.0)
1	25 (73.5)	9 (64.3)	16 (80.0)
2	4 (11.8)	2 (14.3)	2 (10.0)
3	3 (8.8)	2 (14.3)	1 (5.0)
Blinatumomab cycles (%)			
1	20 (58.8)	10 (71.4)	10 (50.0)
2	9 (26.5)	3 (21.4)	6 (30.0)
3	2 (5.9)	1 (7.1)	1 (5.0)
4	3 (8.8)	0 (0.0)	3 (15.0)

NR, non-responders; R=responders, F, female; M, male; R/R, relapsed/Refractory; n, number; IQR, interquartile range (25^th^ percentile - 75^th^ percentile).

### Baseline abundance of circulating CD8^+^ T_EM_, but not surface checkpoint expression, is associated with clinical response to Blinatumomab

**Figure 1:**
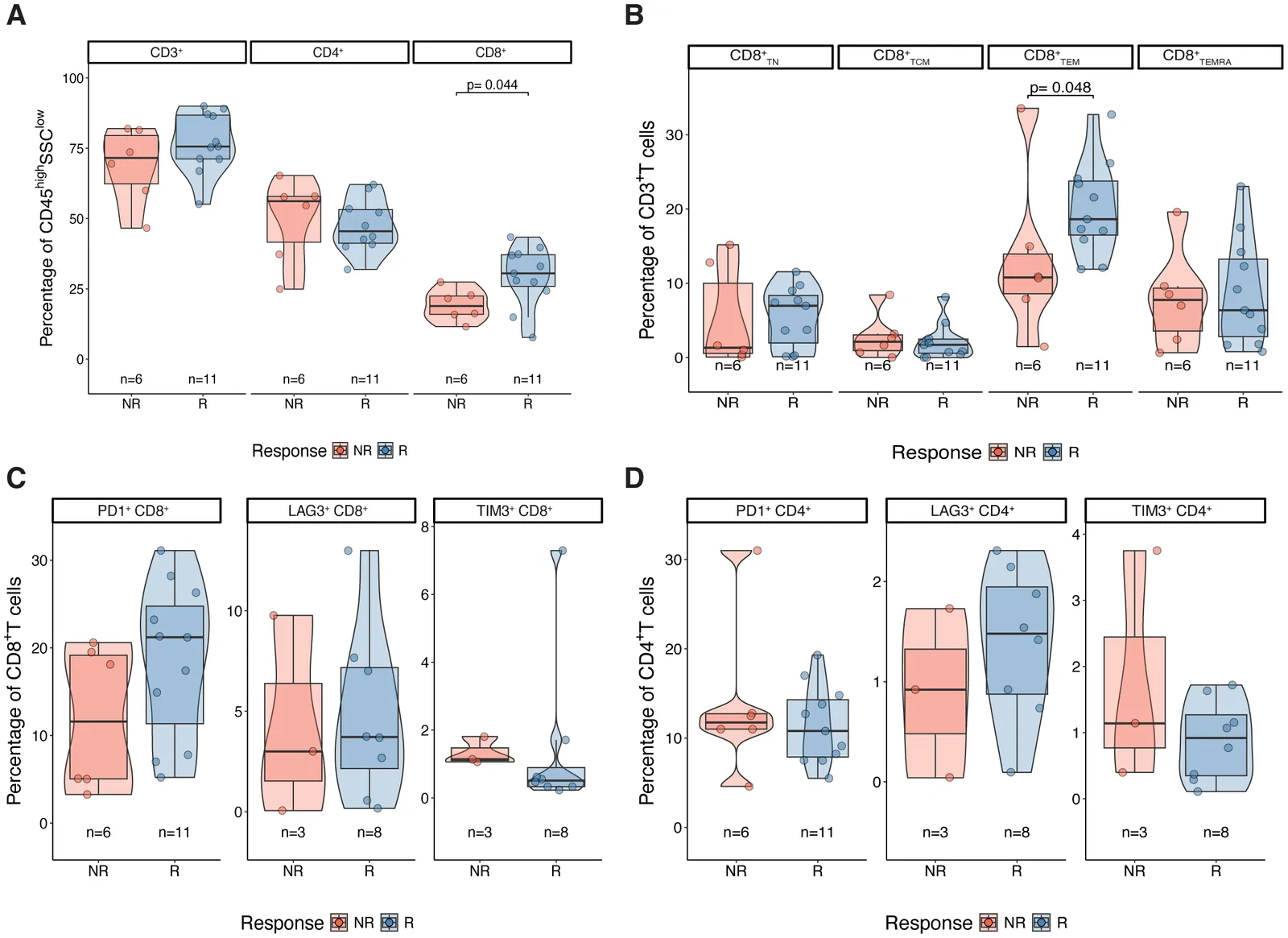
Baseline abundance of circulating CD8+ T_EM_, but not surface checkpoint expression, is associated with clinical response to Blinatumomab. **(A)** Percentage of CD3^+^, CD4^+^, CD8^+^ T cells within CD45^high^SSC^low^ lymphocytes in peripheral blood samples before treatment start; **(B)** Percentage of CD8^+^ Naïve (T_N_), Central Memory (T_CM_), Effector Memory (T_EM_) and Effector Memory expressing CD45RA (T_EMRA_) T cells within CD3^+^ T cells at baseline; **(C)** Percentage of PD1^+^, LAG3^+^, TIM3^+^ CD8^+^ T cells within circulating CD8^+^ T cells at baseline; **(D)** Percentage of PD1^+^, LAG3^+^, TIM3^+^ CD4^+^ T cells within circulating CD4^+^ T cells at baseline. Each box shows 1st quartile, median, 3rd quartile, and whiskers [+/- 1.5*interquartile range (IQR)]. Violin outline width represents density. Each dot represents an individual sample. P-values were calculated using two-sided Wilcoxon rank-sum test.

At baseline, we performed MPFC on 17 patient PBMC samples (R=_-_11, NR=_-_6) to characterize T cell composition. R were characterized by a significantly higher proportion of CD8_+_ T cells among CD45^high^SSC^low^ lymphocytes compared to NR (median 30% vs 19%, p_-_=_-_0.04) (**Figure 1A**). Moreover, R exhibited a higher percentage of CD8^+^ T_EM_ within CD3^+^ T cells (median 18.6% vs 10.9%, p=0.048) (**Figure 1B**). No difference was observed in the baseline proportions of regulatory T cells and CD4^+^ memory subsets between R and NR (**Supplementary Figure 1B**). Interestingly, at baseline, the percentage of CD8^+^ and CD4^+^ cells expressing immune checkpoints (ICs) was not significantly different between R and NR (**Figures 1C, D**). These findings suggest that CD8^+^ T_EM_ may represent the T cell phenotype with the highest sensitivity to Blina-induced activation and cytotoxicity. Conversely, pre-existing T-cell exhaustion may only play a limited role in primary refractoriness.

### Reduced expression of *ZNF683* characterizes the CD8^+^ effector memory compartment in non-responders

**Figure 2:**
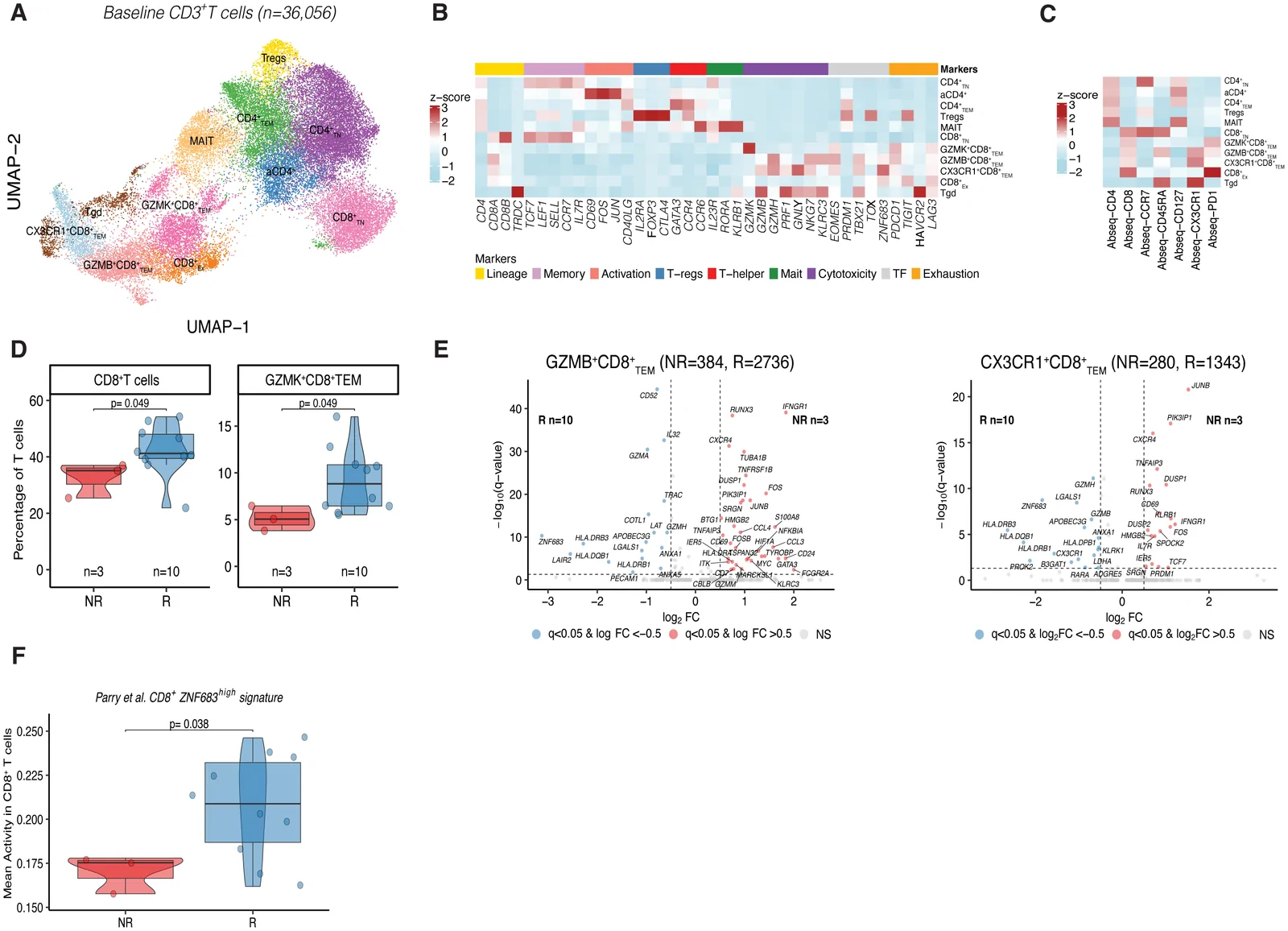
Reduced expression of *ZNF683* characterizes the CD8+ Effector Memory compartment in Non-Responders. **(A)** Uniform Manifold Approximation and Projection(UMAP) embedding of 36,056 CD3^+^ T cells isolated from 13 PBMC samples collected before treatment start; **(B)** Heatmap showing scaled normalized counts of canonical CD4^+^, CD8^+^, memory, and functional gene markers; **(C)** Heatmap showing scaled normalized counts of AbSeq antibody-oligo-conjugates targeting *CD4, CD8, CCR7, CD127, CD45RA, CX3CR1 and PD1.* **(D)** Percentage of CD8^+^ and CD8^+^ T_EM_ GZMK^+^ T cells within each patient CD3^+^ T cells at baseline. Each box shows 1st quartile, median, 3rd quartile, and whiskers (+/- 1.5*interquartile range (IQR)). Violin outline width represents density. Each dot represents an individual sample. P-values were calculated using two-sided Wilcoxon rank-sum test; **(E)** Volcano plot highlighting genes that are highly expressed (log_2_ fold change (log_2_FC)>0.5 & q value<0.05) in NR(n=3) compared to R(n=10) in GZMB^+^CD8^+^ T_EM_(left), and CX3CR1^+^ CD8^+^ T_EM_(right). Vertical dashed lines indicate a |log_2_FC| value of 0.5 and the horizontal dashed line indicate the -log_10_(0.05) threshold. **(F)** Baseline mean activity of a ZNF683^high^ transcriptional module in peripheral CD8^+^ T cells. Violin/boxplots summarize patient-level mean signature activity in R (n=10) versus NR (n=3); each dot represents an individual patient. The P-value was computed using a one-sided Wilcoxon rank-sum test. aCD4^+^ T cells, activated CD4^+^ T cells; CD8^+^ Ex, CD8^+^ Exhausted T cells; MAIT, Mucosal-associated invariant T cells; T_EM_, T Effector Memory T cells; TF, Transcription Factors; Tgd, gamma-delta T cells.

To gain deeper insight into peripheral T cell heterogeneity at baseline, we performed CITE-seq on sorted CD3_+_T cells from a subset of 13 Blina-treated patients (R_-_=_-_10, NR_-_=_-_3). Eleven transcriptionally distinct T cell clusters were identified and annotated using canonical gene and protein markers (**Figures 2A–C**; **Supplementary Table 3**). Consistent with flow cytometry findings, we observed a higher percentage of CD8_+_ T cells within CD3^+^ T cells of R, compared to NR (median 41.2% vs 35.1%, p_-_=_-_0.049; **Figure 2D**). Additionally, R showed higher percentages of *GZMK^+^ CD8*^+^ T_EM_ within CD3^+^ T cells than NR (median value 8.8 vs 5.1%, p=0.048) (**Figure 2D**; **Supplementary Figure 1C**). Compared to other CD8^+^T_EM_ subsets *GZMK+* T cells expressed higher levels of stemness-related genes (*TCF7, LEF1*) and reduced expression of terminal differentiation markers (*KLRG1, CD57, TBX21*), consistent with a higher self-renewal capacity (**Supplementary Figure 1D**, **Supplementary Table 4**). More differentiated *GZMB_+_* and *CX3CR1_+_* CD8_+_T_EM_ displayed marked transcriptional differences between R, and NR. Notably, NR exhibited higher expression of genes associated with T-cell activation, including *CD69, CXCR4, TNFAIP3, and PIK3IP1.* In contrast, R showed upregulation of cytotoxicity-related genes (*GZMA, GZMH, NKG7*) and the cytotoxicity master regulator *ZNF683* (**Figure 2E**). To test whether *ZNF683* upregulation reflected enrichment of a broader cytotoxic transcriptional module, we used a previously described gene expression signature of *ZNF683^high^ CD8*_+_T cells, originally defined in anti–PD-1 responses in Richter transformation (25). Baseline peripheral CD8_+_T cells from R exhibited significantly higher expression of this *ZNF683*-associated program compared with NR (one-sided Wilcoxon p=0.038) (**Figure 2F**; **Supplementary Table 5**).

### Treatment-induced expansion of TIM3+ CD8^+^ T cells drives impairment of Blina-mediated cytotoxicity and correlates with baseline ZNF683 expression

**Figure 3:**
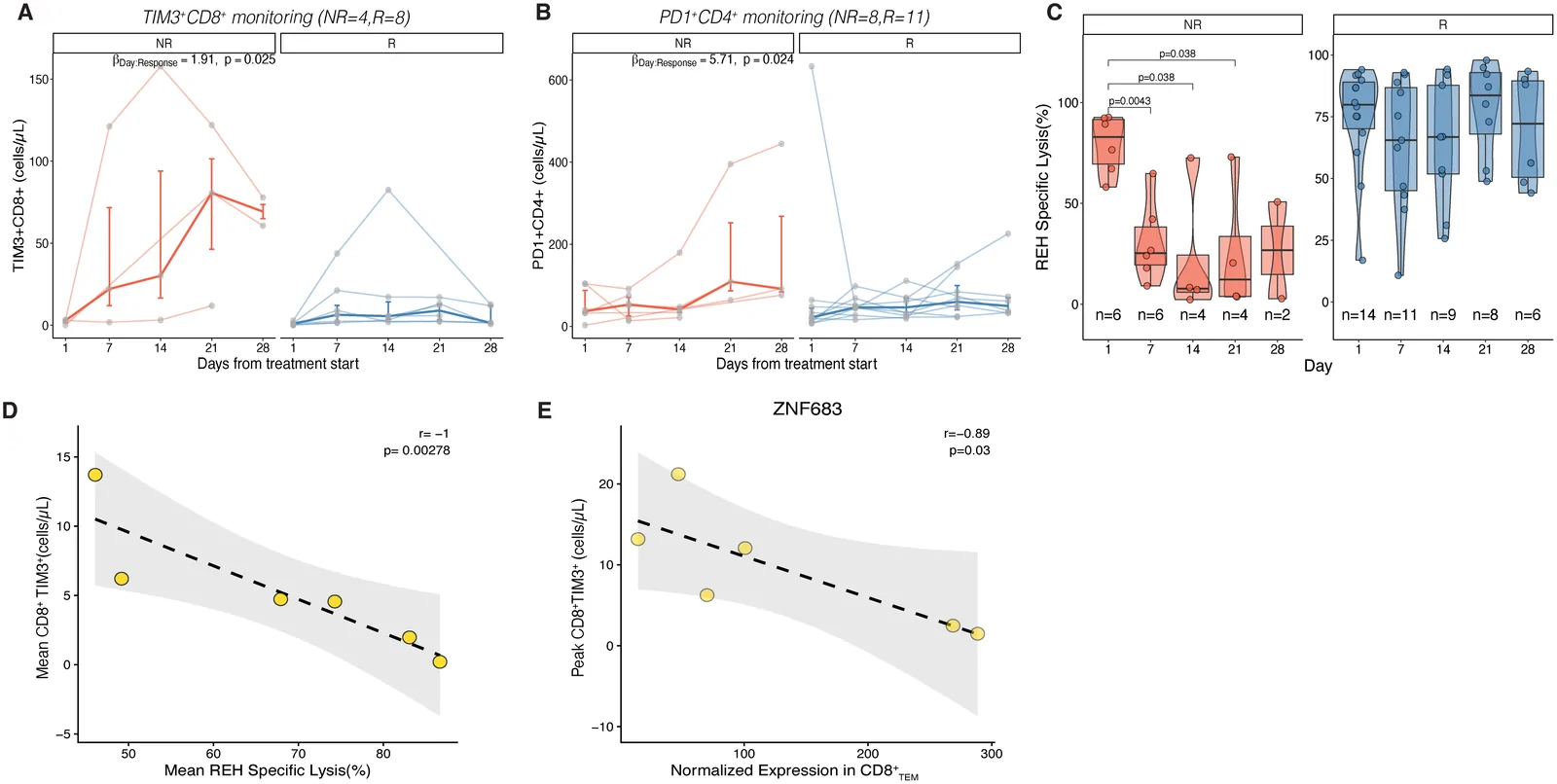
Treatment-induced expansion of TIM3+ CD8+ T cells drives impairment of Blina-mediated cytotoxicity and correlates with baseline ZNF683 expression. **(A,B)** Absolute counts of TIM3^+^CD8^+^ and PD1^+^ CD4^+^ T cells measured weekly through the first cycle of blinatumomab. Each dot represents a sample and samples from the same patient are connected by lines. Median values and interquartile ranges in R and NR are shown by bold lines (blue=R, red=NR). **(C)** *Ex-vivo* REH cell line-specific lysis measured weekly during the first cycle of blinatumomab treatment. Each box shows 1st quartile, median, 3rd quartile, and whiskers (+/- 1.5*interquartile range (IQR)). Violin outline width represents density. Each dot represents an individual sample. P-values were calculated using two-sided Wilcoxon rank-sum test; **(D)** Scatterplots showing the correlation between the peak absolute value of TIM3^+^CD8^+^ T cells during the treatment and *ex-vivo* REH specific lysis in 6 patients. Each dot represents a patient, the dashed line indicates a linear regression fit, and the grey area represents the 95% confidence interval. Spearman’s correlation coefficient (r) and p-value are shown. The average values of REH specific lysis were used to account for repeated measurements. **(E)** Scatterplot showing the correlation between the aggregated and normalized expression value of ZNF683 in CD8^+^ T_EM_ at baseline and the peak absolute value of CD8^+^TIM3^+^ T cells observed during treatment. Each dot represents a patient, the dashed line indicates a linear regression fit, and the grey area represents the 95% confidence interval. Spearman’s correlation coefficient (r) and p-value are shown.

To characterize the longitudinal evolution of T-cell responses during the first treatment cycle, we applied linear mixed-effects models (LMMs) to longitudinal flow cytometry data (**Supplementary Table 6**). Interestingly, compared to R (n=8), NR (n=4) showed a significantly higher increase in the absolute counts of TIM3^+^CD8^+^T cells (β timexresponse: 1.91, p=0.025) (**Figure 3A**; **Supplementary Table 7**) and of PD1^+^CD4^+^T cells (β timexresponse: 5.71, p=0.024) (**Figure 3B**) over time. To determine the functional consequence of this phenotypic shift, we performed longitudinal *ex-vivo* cytotoxicity assays. NR underwent an early and progressive collapse in T-cell function, with Blinatumomab-mediated lysis reaching its nadir at Day 14 (median specific lysis Day 14 7.77% vs 83% at Day1, p=.038) (**Figure 3C**). To investigate the potential contribution of T-cell exhaustion to the observed decline in T cell function, we analyzed the subgroup of patients for whom both flow cytometry and functional data were available. Crucially, we found that patients with higher average absolute count of TIM3^+^CD8^+^T cells during treatment were characterized by lower average *ex-vivo* T cell cytotoxicity (Spearman’s R= –1, p=0.0027) (**Figure 3D**). In contrast, no such correlation was found for PD1^+^CD4^+^T cells, identifying the TIM3^+^CD8^+^ subset as the key driver of functional decline (**Supplementary Figure 1E**). Lastly, to investigate if baseline transcriptional features of CD8^+^T_EM_ might contribute to treatment-induced T-cell exhaustion, we integrated CITE-seq and longitudinal flow cytometry data from 6 patients. Interestingly, the peak absolute cell count per µl of TIM3^+^CD8^+^T cells during treatment was negatively correlated with *ZNF683* expression (R=-0.89, p=.03) (**Figure 3E**) in baseline CD8^+^T_EM_ cells.

### Circulating markers of M2-polarization and checkpoint signaling correlate with treatment-induced T-cell exhaustion

**Figure 4:**
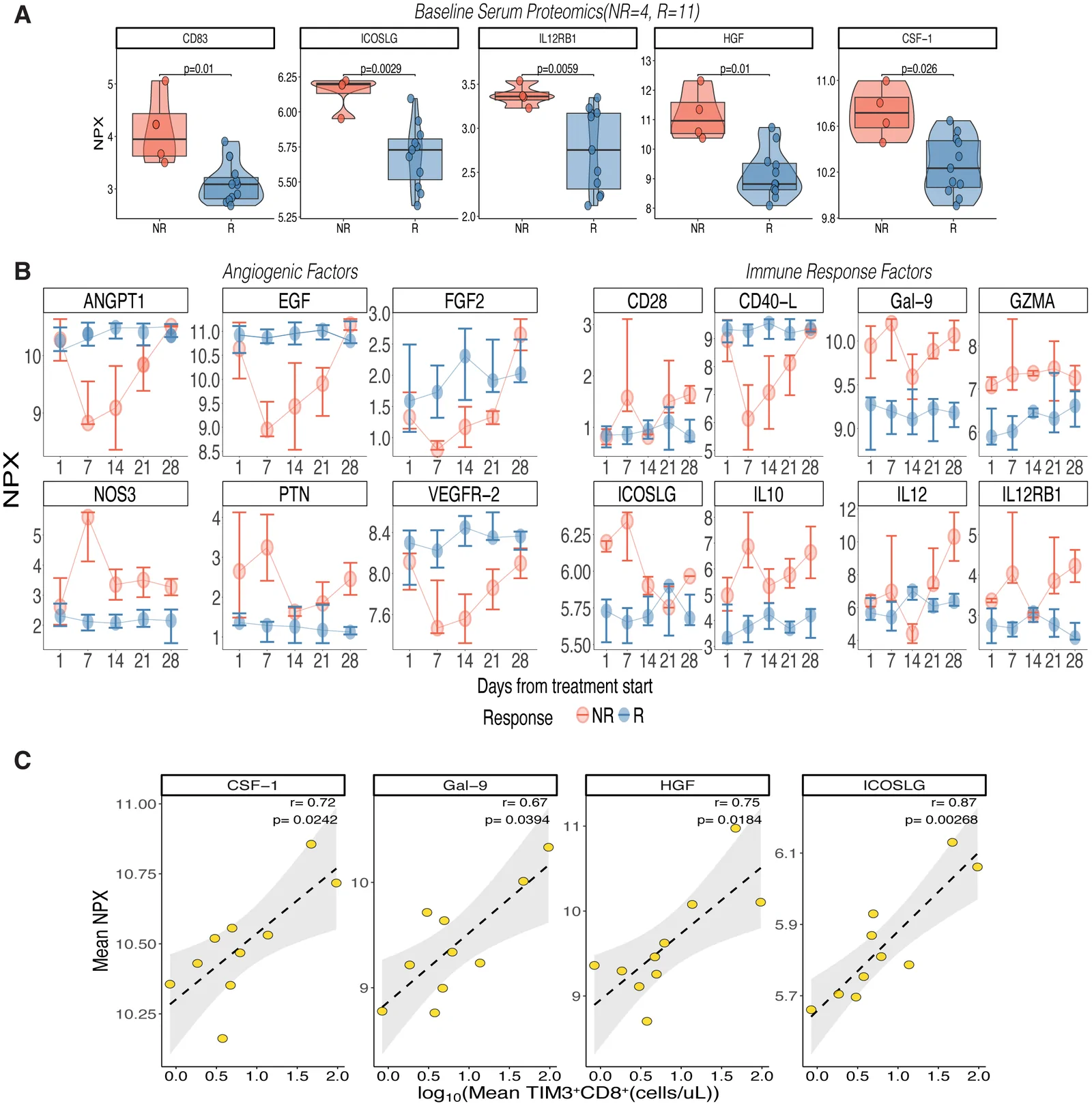
Circulating markers of M2-polarization and checkpoint signaling correlate with treatment-induced T-cell exhaustion. **(A)** Pre-treatment NPX levels of proteins that were significantly deregulated at baseline between R and NR. Each box shows 1st quartile, median, 3rd quartile, and whiskers (+/- 1.5*interquartile range (IQR)). Violin outline width represents density. Each dot represents an individual sample. P-values were calculated using two-sided Wilcoxon rank-sum test; **(B)** Line graph showing NPX medians and interquartile ranges of proteins with significant differences between R and NR at any timepoint during treatment. **(C)** Scatterplots showing the correlation between the average absolute value of TIM3^+^ CD8^+^ and the average NPX value during treatment. Each dot represents a patient, the dashed line indicates a linear regression fit, and the grey area represents the 95% confidence interval. Spearman’s correlation coefficient (r) and p-value are shown.

To identify soluble biomarkers of response and contributors to the observed post-treatment T-cell exhaustion, we profiled the serum proteome of 17 patients at multiple time points. Linear mixed-effects models identified 21 proteins whose levels were significantly associated with response, treatment dynamics, or their interaction (**Supplementary Table 8**). Specifically, at baseline, NR possessed significantly elevated levels of CSF-1, HGF, and ICOSLG compared to R (**Figure 4A**). As CSF-1 and HGF are canonical key regulators of M2-like macrophage polarization, their elevation suggests a pre-existing environment primed for myeloid-mediated suppression. Longitudinally, NR were characterized by rising levels of the TIM-3 ligand Galectin-9 (Gal-9) and IL-10, alongside a reduction in angiogenic factors (**Figure 4B**; **Supplementary Figure 1F**). Notably, we also observed a trend toward higher Gal-9 levels in NR at baseline (p=0.07), indicating both static and dynamic involvement.

To investigate the relationship between these factors and the treatment-induced T-cell exhaustion, we integrated our longitudinal proteomic and flow cytometry data (n patients=10). Strikingly, the average circulating levels of CSF-1, HGF, ICOSLG, and Gal-9 showed strong positive correlations with the mean concentration of TIM3_+_CD8_+_T cells (**Figure 4C**; **Supplementary Table 9**), supporting their role as potential contributors to Blina-induced T-cell exhaustion.

## Discussion

Blina represents a cornerstone for treating patients with relapsed/refractory and MRD^+^ BCP-ALL. However, refractoriness and secondary relapses represent an unmet medical need, and immunological and molecular mechanisms underpinning the lack of response remain unclear. Building on our previous finding that continuous Blina exposure drives T-cell exhaustion, this study identifies baseline T-cell states and systemic immune-microenvironmental determinants that shape exhaustion trajectories and therapeutic failure. By leveraging a high-resolution, multi-omic approach in 34 patients, we identified significant compositional and transcriptional differences in the baseline peripheral T-cell landscape. Redistribution of circulating T cells to the bone marrow niche on the first day of treatment underscores that peripheral blood T cells play a crucial role in response to Blina (26–28). Here, we show that a higher proportion of CD8^+^T_EM_ is associated with clinical responses, consistent with pre-clinical studies showing that CD8^+^T_EM_ are the memory subset preferentially activated by Blina (29). In-depth transcriptional profiling identified a specific *GZMK*^+^*CD8*^+^ T_EM_ subset enriched at baseline in R. Compared to other T_EM_ subsets, these cells displayed a less terminally differentiated transcriptional profile, with higher expression of stemness-associated genes such as *TCF7* and *LEF1*, consistent with a precursor state that retains proliferative capacity and may serve as a renewable source of cytotoxic effectors under continuous CD3 stimulation. This aligns with recent evidence positioning *GZMK^+^*T_EM_ as a less differentiated precursor within the CD8^+^ effector memory compartment (30) and with their association with favorable outcomes to immunotherapies in both solid (31, 32) and hematological malignancies (33, 34).

Crucially, multi-omic profiling revealed that baseline surface expression of PD-1, TIM-3, and LAG-3 on peripheral T cells was comparable between R and NR, suggesting that pre-treatment T-cell exhaustion may play a limited role in primary resistance to Blina. Alternatively, we observed that CD8^+^T_EM_ of NR displayed significant upregulation of T-cell activation genes without proportionate cytotoxic commitment at baseline. In contrast, pre-treatment CD8^+^T_EM_ of R showed increased expression of cytotoxicity-related genes together with higher levels of *ZNF683*. *ZNF683* encodes the transcription factor Hobit, which is critical for maintaining cytotoxic competence in CD8^+^memory T cells (35), and its deficiency signals a molecular vulnerability. This mirrors recent findings in Richter transformation and AML, where *ZNF683* expression defined the specific T-cell subsets responsible for superior clinical responses to PD-1 blockade and allogeneic stem cell transplantation, respectively (25, 36).

Importantly, these baseline transcriptional features were associated with divergent T-cell differentiation trajectories following Blina administration. NR displayed a pronounced post-treatment expansion of TIM3_+_CD8_+_T cells in peripheral blood, which coincided with a progressive decline in ex vivo T-cell cytotoxicity. Notably, the magnitude of TIM3_+_CD8^+^exhausted T-cell expansion during treatment inversely correlated with *ZNF683* expression, suggesting that the pre-treatment transcriptional state of CD8_+_T_EM_ shapes post-treatment T-cell exhaustion in response to Blina. Integrative analysis of longitudinal serum proteomics and cellular immunophenotyping revealed that also circulating levels of CSF-1, HGF, ICOSLG, and galectin-9 strongly correlated with the magnitude of TIM3_+_CD8_+_T-cell expansion during treatment. The association with Galectin-9, the canonical ligand of TIM-3 (37–39), supports a model in which extrinsic inhibitory signaling, potentially originating from leukemic blasts (40), cooperates with intrinsic T-cell vulnerabilities to drive T-cell exhaustion under sustained CD3 engagement. In parallel, elevated baseline levels of CSF-1 and HGF in NR point to a pre-existing systemic milieu skewed toward M2-like macrophages, which have been described to actively interact with tumor-infiltrating CD8_+_T cells and foster the development of T-cell exhaustion (41–43).

To our knowledge this is the largest study integrating multi-omic single-cell, proteomic and functional data to investigate cellular and molecular mechanisms of resistance to Blina to date. While the relatively small number of patients in some subgroup analyses, particularly the Non-Responder group in our CITE-seq cohort (n=3), remains a limitation, our data provides a rationale for novel risk stratification and potentially transformative combinatorial strategies, and these findings warrant further validation in larger, independent cohorts that also account for disease state at baseline (R/R versus MRD-positive), given that these represent clinically and potentially biological distinct settings. *ZNF683* expression defines a cytotoxic–programmed T-cell state and may serve as a baseline predictive biomarker. Targeting the myeloid compartment (e.g., via CSF-1R inhibition) or blocking the Gal-9/TIM-3 could remodel the suppressive milieu and prevent the terminal exhaustion that currently limits the response to Blina. In conclusion, these molecular insights advance the potential for personalized immunotherapy in BCP-ALL, and potentially also for other T cell recruiting antibodies, by defining the systemic and intrinsic determinants of T-cell fate.

## Data Availability

The datasets presented in this study can be found in online repositories. The names of the repository/repositories and accession number(s) can be found below: https://www.ncbi.nlm.nih.gov/geo/, GSE314616 - token: udmdiwomdfwhxol.

## Supplementary material

Supplementary Figure 1 **(A)** Venn diagram illustrating the overlap of patients across four assay platforms: Multiparametric Flow Cytometry (MPFC, n=19), Ex-Vivo Cytotoxicity (n=26), CITE‐seq (n=13), and Serum Proteomics (n=17). The intersecting areas denote patients analyzed with multiple techniques during the treatment course; **(B)** Percentage of T-regs, CD4^+^ Naïve (T_N_), Central Memory (T_CM_), Effector Memory (T_EM_) and Effector Memory expressing CD45RA (T_EMRA_) T cells within CD3+ T cells at baseline, assessed by flow cytometry. Each box shows 1st quartile, median, 3rd quartile, and whiskers (+/- 1.5*interquartile range (IQR)). Violin outline width represents density. Each dot represents an individual sample. P-values were calculated using two-sided Wilcoxon rank-sum test; **(C)** Percentage of transcriptionally-distinct CD3+ T-cell clusters within each baseline peripheral T cell sample from 13 patients(NR = 3,R=10). Each box shows 1st quartile, median, 3rd quartile, and whiskers (+/- 1.5*interquartile range (IQR)). Violin outline width represents density. Each dot represents an individual sample. P-values were calculated using two-sided Wilcoxon rank-sum test; **(D)** Dot plot showing expression patterns of memory-associated and terminal differentiation genes across CD8_+_ effector memory T-cell subsets at baseline. Color intensity represents the average scaled gene expression within each cluster, while dot size indicates the percentage of cells expressing each gene. **(E)** Scatterplot showing the correlation between the peak absolute value of PD1^+^CD4^+^ T cells and ex-vivo REH specific lysis in 6 patients. Each dot represents a patient, the dashed line indicates a linear regression fit, and the grey area represents the 95% confidence interval. Spearman’s correlation coefficient (r) and p-value are shown. The average values of REH specific lysis were used to account for repeated measurements; **(F)** Dotplot and lineplot showing the difference in median NPX values between NR (right side) and R (left side) at each available timepoint for each protein. The 21 proteins showed in the plot were selected due to significant association with response, time, and/or interaction effects. Each panel corresponds to one time point, with proteins grouped by functional class (Angiogenic vs Immune response-related proteins). P-values were calculated using two-sided Wilcoxon rank-sum test and dots are colored by -log10(p-values). Dots indicating statistically significant differences (p-value<0.05 and q-value<0.25) are triangle-shaped. NPX = Normalized Protein eXpression.
