# Supplementary material for: Pre-treatment T cell features and immune-milieu characteristics shape treatment-induced exhaustion and resistance to Blinatumomab in B-cell acute lymphoblastic leukemia
Source: Front Immunol. 2026 Aug 20;17:1895742. doi: 10.3389/fimmu.2026.1895742 (PMC13538086; doi:10.3389/fimmu.2026.1895742)
Supplement: Supplementary file 1 [file DataSheet1.pdf]

Supplementary Table 2. Patient characteristics.

Patient	Age (years)	Sex	Disease state	Response	Blinatumomab cycles (n)	Previous therapies (n)	Most recent therapy prior to blinatumomab	BM blasts pre-Blina (%)	BM blasts post-Blina (%)	MRD level post-Blina	Prior CAR-T	CiTE-seq	MPFC	Olink	Functional ex-vivo assay	T cells (CiTE-seq, n)
1	71.0	F	R/R	R	2	1	Cyclophosphamide/Dexamethasone	88	<5	NA	No	No	No	Yes	Yes	N/A
2	68.0	F	R/R	R	2	1	Induction chemotherapy	24	<5	NA	No	Yes	Yes	No	Yes	3,937
3	33.5	F	R/R	R	2	1	No therapy for r/r disease	70	<5	NA	No	No	No	No	Yes	N/A
4	56.6	M	R/R	R	2	2	FLAG-Ida	60	<5	NA	No	No	No	No	Yes	N/A
5	23.3	M	R/R	R	1	3	HyperCVAD	92	<5	$<1\times10^{-5}$ (positive)	No	No	No	No	Yes	N/A
6	36.7	M	R/R	NR	1	1	Vincristine/Dexamethasone	54	98	NA	No	No	No	No	Yes	N/A
7	36.4	M	R/R	NR	1	3	Vincristine	88	99	NA	No	No	No	No	Yes	N/A
8	51.8	M	R/R	NR	1	1	Cyclophosphamide	40	46	NA	No	No	Yes*	No	Yes	N/A
9	42.6	F	R/R	NR	2	1	Induction chemotherapy	12	Extramedullary relapse	NA	No	No	No	No	Yes	N/A
10	30.1	M	R/R	NR	1	1	Re-induction chemotherapy	49	50	NA	No	No	No	No	Yes	N/A
11	34.8	M	R/R	NR	1	1	No therapy for r/r disease	45	Extramedullary relapse	NA	No	No	No	No	Yes	N/A
12	20.4	M	R/R	NR	1	2	No therapy for r/r disease	6	70	NA	No	Yes	Yes	No	Yes	2,883
13	72.6	M	R/R	NR	1	1	Cyclophosphamide	39	82 (PB)	NA	No	Yes	Yes	Yes	Yes	3,283
14	69.4	M	R/R	NR	1	2	TKI	77	20	NA	No	No	Yes	Yes	No	N/A
15	45.7	M	MRD+	R	1	1	Consolidation chemotherapy	<5	<5	Negative	No	Yes	No	No	Yes	2,255
16	34.3	M	MRD+	R	3	1	Consolidation chemotherapy	<5	<5	Negative	No	No	Yes	No	Yes	N/A
17	71.5	F	MRD+	R	4	1	Consolidation chemotherapy	<5	<5	Negative	No	No	No	No	Yes	N/A
18	28.4	F	MRD+	R	1	1	Consolidation chemotherapy	<5	<5	Negative	No	Yes	Yes	Yes	Yes	2,424
19	62.1	M	MRD+	R	4	1	Consolidation chemotherapy	<5	<5	Negative	No	Yes	Yes	Yes	Yes	3,172
20	34.0	M	MRD+	R	1	1	HyperCVAD	<5	<5	Negative	No	Yes	Yes	Yes	Yes	2,228
21	28.0	M	MRD+	R	2	2	Consolidation chemotherapy	<5	<5	Negative	No	Yes	Yes	Yes	Yes	2,204
22	26.0	F	MRD+	R	1	1	Consolidation chemotherapy	<5	<5	Negative	No	Yes	No	Yes	Yes	2,821
23	52.4	M	MRD+	R	1	1	Consolidation chemotherapy	<5	<5	Negative	No	Yes	Yes	Yes	Yes	1,801
24	19.4	M	MRD+	R	1	1	Consolidation chemotherapy	<5	<5	Negative	No	Yes	Yes	Yes	Yes	3,020
25	26.6	M	MRD+	R	2	1	Consolidation chemotherapy	<5	<5	Negative	No	Yes	Yes	Yes	Yes	3,911
26	55.2	M	MRD+	R	1	1	Consolidation chemotherapy	<5	<5	Negative	No	No	Yes	Yes	No	N/A
27	31.6	M	MRD+	R	1	1	Consolidation chemotherapy	<5	<5	Negative	No	No	No	Yes	No	N/A
28	75.7	F	MRD+	R	4	0	Induction chemotherapy	<5	<5	Negative	No	No	Yes	No	No	N/A
29	24.2	F	MRD+	R	1	1	Consolidation chemotherapy	<5	<5	Negative	No	No	No	Yes	No	N/A

Patient	Age (years)	Sex	Disease state	Response	Blinatumomab cycles (n)	Previous therapies (n)	Most recent therapy prior to blinatumomab	BM blasts pre-Blina (%)	BM blasts post-Blina (%)	MRD level post-Blina	Prior CAR-T	CiTE-seq	MPFC	Olink	Functional ex-vivo assay	T cells (CiTE-seq, n)
30	59.8	M	MRD+	NR	3	1	Maintenance therapy following induction & consolidation	<5	<5	1×10^{-3}	No	No	Yes	No	Yes	N/A
31	38.2	M	MRD+	NR	2	1	Consolidation chemotherapy	<5	<5	Positive ($<1\times10^{-3}$)	No	Yes	Yes	Yes	Yes	2,117
32	71.7	F	MRD+	NR	1	0	Induction chemotherapy	<5	10	2×10^{-1}	No	No	Yes	Yes	No	N/A
33	37.0	M	MRD+	NR	2	1	Consolidation chemotherapy	<5	<5	2×10^{-3}	No	No	Yes	No	No	N/A
34	23.2	F	MRD+	NR	1	3	Consolidation chemotherapy + TKI	<5	<5	BCR::ABL1 ratio 0.446	No	No	Yes	Yes	No	N/A

**Patient no.8: included in MPFC analysis but removed from downstream analyses due to absolute $CD3^+$ T cell count <30 at each timepoint.*

BM, bone marrow; Blina, blinatumomab; MRD, minimal residual disease; CAR-T, chimeric antigen receptor T cell; CiTE-seq, cellular indexing of transcriptomes and epitopes by sequencing; MPFC, multiparameter flow cytometry; NA, not applicable; NR, non-responder; PB, peripheral blood; R, responder; R/R, relapsed/refractory; TKI, tyrosine kinase inhibitor.
